# Specific circulating microRNA signature of bicuspid aortic valve disease

**DOI:** 10.1186/s12967-017-1176-x

**Published:** 2017-04-11

**Authors:** Neus Martínez-Micaelo, Raúl Beltrán-Debón, Isabel Baiges, Marta Faiges, Josep M. Alegret

**Affiliations:** 1 Grup de Recerca Cardiovascular, Institut d’Investigació Sanitària Pere Virgili (IISPV), Universitat Rovira i Virgili, Reus, Spain; 2 Centre for Omic Sciences (COS), Universitat Rovira i Virgili, Reus, Spain; 3 Servei de Cardiologia, Hospital Universitari de Sant Joan, Departament de Medicina i Cirurgia, Universitat Rovira i Virgili, c/Dr Josep Laporte, 1, 43204 Reus, Spain

**Keywords:** microRNA, Bicuspid aortic valve, Aortic dilation

## Abstract

**Background:**

We aimed to determine the circulating miRNA expression profile associated with BAV and aortic dilation to provide diagnostic and prognostic biomarkers for BAV and/or aortic dilation.

**Methods and results:**

We applied a miRNome-wide microarray approach using plasma samples (n = 24) from healthy tricuspid aortic valve individuals, BAV patients and BAV patients with aortic dilation to compare and identify the specific miRNAs associated with BAV and aortic dilation. In a second stage, the expression patterns of the miRNA candidates were validated by RT-qPCR in an independent cohort (n = 43). The miRNA microarray data and RT-qPCR analyses revealed that the expression levels of circulating miR-122, miR-130a and miR-486 are significantly influenced by the morphology of the aortic valve (bicuspid/tricuspid) and could be functionally involved in the regulation of TGF-β_1_ signalling. Furthermore, the expression pattern of miR-718 in the plasma was strongly influenced by dilation of the ascending aorta. miR-718 expression was inversely correlated with the aortic diameter (R = −0.63, p = 3.1 × 10^−5^) and was an independent predictor of aortic dilation (β = −0.41, p = 0.022). The genes targeted by miR-718 are involved in the regulation of vascular remodelling.

**Conclusions:**

We propose that miR-122, miR-130a, miR-486 and miR-718 are new molecular features associated with BAV and aortic dilation principally by the activation of TGF-β_1_ pathway and vascular remodelling mediated by VEGF signalling pathways.

## Background

Bicuspid aortic valve (BAV) represents the most common congenital cardiac malformation and is commonly associated with the development of aortic valve dysfunction and the progressive dilation of the ascending aorta. In patients with BAV, the dilation of the aorta is associated with aortic regurgitation [1] and the risk of aortic dissection and thus often requires prophylactic aortic surgery [2–6]. The causes of aorta dilation have been debated for several years, and changes in the flow characteristics within the ascending aorta have been suggested as the main cause [7, 8]. However, the mechanisms involved have not been fully elucidated.

MicroRNAs (miRNAs) are endogenously expressed 19–23nt-long noncoding RNAs (ncRNAs) that silence gene expression at the post-transcriptional level, largely via imperfect base-pairing interactions that occur preferentially within the 3′ untranslated regions (UTRs) of target mRNAs. Due to the ability of miRNAs to target hundreds of mRNAs, they are considered as potent post-transcriptional regulators, and in fact, it is suggested that a significant portion of the human genome is regulated by miRNAs. Aside from their intracellular function, miRNAs can be exported or released by cells into the circulating blood in highly stable forms [9]. miRNA signatures have been proposed as potential biomarkers with the potential to improve disease diagnosis and prognosis in clinical practice and have been identified as useful biomarkers for a wide range of cardiovascular diseases, including cardiovascular calcification [10], vulnerable coronary artery disease [11], heart failure [12], myocardial infarction [13], abdominal aortic aneurysm [14] and coronary artery disease [15]. Despite the large number of miRNAs identified thus far, the biological roles of most miRNAs and the molecular mechanisms underlying their function remain largely unknown.

In this study, we applied a miRNome-wide microarray approach using the plasma of healthy tricuspid aortic valve (TAV) individuals and BAV patients [with or without aortic dilation (BAV_dil_)] to compare and identify specific miRNAs associated with BAV and the dilation of the ascending aorta. In a second stage, the expression levels of the miRNA candidates were validated in an independent cohort, providing new insights into the complex mechanisms that underlie BAV and aortic dilation.

## Methods

### Study population

The participants included in this study belong to a cohort of prospective patients with BAV that were followed-up in our facilities. Upon enrolment, the participants were prospectively entered into a specific database, underwent a blood draw and provided informed written consent. The samples were stored until needed in our biological sample bank (Biobanc IISPV-HUSJR). BAV diagnosis was made when two aortic leaflets were clearly visualized, with or without a raphe, either on the parasternal short-axis view of a transthoracic echocardiogram [16], on a transesophageal echocardiogram [16] or on a cardiac magnetic resonance [17]. Explorations were performed or supervised by the same observer (JMA). This study was approved by the Institutional Review Board (the Clinical Ethics Committee) of our institution.

The study design was composed of two evaluations to determine the possible miRNAs associated with BAV and aortic dilation. First, we studied the association of BAV with the modulation of the circulating miRNOme profile by determining the miRNA expression profile in the plasma of patients diagnosed with BAV (BAV_non-dil_; n = 9) or BAV and aortic dilation (BAV_dil_; n = 9) using microarrays, and then, we compared these with the expression profiles of healthy TAV control subjects (TAV_non-dil_; n = 6). For this stage and to improve the study power, we selected three groups composed of patients with characteristics that were extremely homogeneous (Table 1) to exclude possible confounding factors, such as age, sex or BMI. In addition, patients with aortic stenosis, hypertension, diabetes mellitus or pharmacologic treatment (including statins, ACE/ARII and/or β-blockers) were excluded. An aortic diameter lower than 19 mm/m^2^ or higher than 21 mm/m^2^ was used as the criterion to differentiate between the non-dilated and dilated patients, respectively.

**Table 1:** Clinical and echocardiographic characteristics of the patients included in the microarray

	Control	BAV	BAVdil	p
Age (years)	41 ± 4	33 ± 3	41 ± 4	0.177
Sex (male/female)	(6/0)	(6/0)	(6/0)	1.000
Body weight (kg)	74.2 ± 2.2	75.0 ± 4.2	79.0 ± 5.7	0.743
Hypertension	0 (0%)	0 (0%)	0 (0%)	1.000
Hypercholesterolemia	0 (0%)	0 (0%)	2 (22.2%)	0.162
Smoker	1 (20.0%)	2 (22.2%)	3 (33.3%)	0.814
Aortic stenosis (mean gradient ≥20 mm Hg)	0 (0%)	0 (0%)	0 (0%)	1.00
Aortic regurgitation (≥II)	0 (0%)	3 (33.3%)	6 (66.7%)	0.031*
Aortic valve gradient (mean, mm Hg)	3.75 ± 0.3	4.6 ± 0.8	7.22 ± 1.6	0.190
Left ventricle diastolic diameter (mm)	51.67 ± 1.5	50.75 ± 1.0	55.11 ± 1.3	0.050
Left ventricle systolic diameter (mm)	32.33 ± 0.9	31.88 ± 2.7	33.78 ± 1.4	0.484
Left ventricular ejection fraction (%)	74.40 ± 3.2	71.25 ± 1.2	71.33 ± 2.6	0.641
Ascending aorta diameter (mm/m^2^)	15.05 ± 0.4	15.74 ± 0.7	20.71 ± 1.1	0.001**

*BAV* bicuspid aortic valve patients, *BAVdil* patients with bicuspid aortic valve and aortic dilation

* Significant values (p < 0.05)

** Significant values (p < 0.01)

In the second evaluation, we validated the miRNA candidates by RT-qPCR in a new cohort of individuals comprising TAV (n = 19) and BAV individuals (n = 24) (Table 2). The BAV group comprised BAV_non-dil_ (n = 12) and BAV_dil_ (n = 12) patients. The TAV group comprised healthy controls TAV_non-dil_ (n = 12), and we also included a new group of patients with TAV and aortic dilation (TAV_dil_; n = 7) in this evaluation to determine the specific effects associated with valve morphology or aortic dilation per se. Patients with Marfan syndrome were excluded.

**Table 2:** Clinical and echocardiographic characteristics of the independent cohort used in the validation study

	TAV	TAVdil	BAV	BAVdil	p
Age (years)	39 ± 3	65 ± 3	33 ± 3	42 ± 3	<0.001**
Sex (male/female)	(8/4)	(5/2)	(11/1)	(6/6)	0.171
Body weight (kg)	70.1 ± 3.7	76.8 ± 3.4	73.25 ± 4.4	66.6 ± 4.2	0.374
Hypertension	0 (0%)	5 (71.4%)	1 (8.3%)	3 (25%)	0.002**
Hypercholesterolemia	1 (8.3%)	1 (14.3%)	0 (0%)	0 (0%)	0.391
Smoker	0 (0%)	0 (0%)	1 (8.3%)	3 (25%)	0.105
Aortic stenosis (mean gradient ≥20 mm Hg)	0 (0%)	0 (0%)	1 (8.3%)	2 (16.7%)	0.507
Aortic regurgitation (≥II)	0 (0%)	4 (57.1%)	7 (46.7.3%)	4 (33.3%)	0.012*
Aortic valve gradient (mean, mm Hg)	3.4 ± 0.2	3.0 ± 0.1	6.7 ± 1.0	16.5 ± 5.8	0.117
Left ventricle diastolic diameter (mm)	50.1 ± 1.2	53.0 ± 2.2	54.8 ± 2.0	52.3 ± 0.9	0.182
Left ventricle systolic diameter (mm)	29.9 ± 0.7	35.8 ± 2.9	34.7 ± 0.7	32.2 ± 0.7	0.048*
Left ventricular ejection fraction (%)	77.3 ± 1.6	61.0 ± 11.7	69.1 ± 2.6	70.7 ± 1.2	0.063
Ascending aorta diameter (mm/m^2^)	15.9 ± 0.6	23.2 ± 0.9	17.6 ± 0.8	25.1 ± 1.2	<0.001**

*BAV* bicuspid aortic valve patients, *BAVdil* patients with bicuspid aortic valve and aortic dilation

* Significant values (p < 0.05)

** Significant values (p < 0.01)

### Blood sampling

Blood samples were collected in overnight fasting conditions in the early morning and were processed within 90 min after collection. The samples were centrifuged at 1500*g* for 15 min to obtain plasma. The plasma samples were stored at −80 °C in our biological sample bank until needed (Biobanc IISPV-HUSJR).

### RNA extraction and miRNA microarray preparation

Total RNA was extracted from 250 μL of plasma using the TRIzol reagent according to the manufacturer’s instructions (Invitrogen) and purified using an RNeasy minikit (Qiagen). To increase RNA recovery, 1 μg of MS2 carrier RNA was added to each plasma sample. The quality of the total RNA isolated was determined using an Agilent 2100 Bioanalyser.

The plasma miRNA expression levels for 24 individuals were assessed using SurePrint G3 human 8 × 60 k miRNA microarrays (Agilent Technologies), covering 1205 human miRNAs (Sanger miRBase release 16). The miRNAs were dephosphorylated and labelled with cyanine 3-cytidine biphosphate, including a labelling spike-in solution (Agilent Technologies) to assess the labelling efficiency. The samples were hybridized on the arrays, including a hybridization spike-in solution (Agilent Technologies) to monitor the hybridization efficiency. The arrays were scanned with a G2565CA Microarray Scanner System with SureScan High Resolution Technology (Agilent Technologies) using Scan Control software. The Feature Extraction 11.5.11 (Agilent Technologies) and GeneSpring 12.6.1 software packages were used for data processing, and all steps were performed according to the manufacturer’s protocol.

### Microarray analysis of the miRNA expression data

The data from microarrays were normalized using the robust multi-array average (RMA) method [18], implemented in the AgiMicroRna Bioconductor package, and the fold changes of the circulating miRNAs were determined using the linear model implemented in the limma Bioconductor package [19]. The Benjamini and Hochberg method was used to adjust the p values for multiple testing and to control the false discovery rate [20].

### MicroRNA quantification by real-time qRT-PCR

The microarray expression data were validated in an independent patient cohort (*n* = 43), including healthy TAV controls, BAV patients, BAVdil patients, and an additional group of TAV individuals with aortic dilation. Reverse transcription was performed using the TaqMan MicroRNA Reverse Transcription Kit (Applied Biosystems) and the miRNA-specific reverse-transcription primers provided with the TaqMan MicroRNA Assay (Applied Biosystems) to analyse the expression of each miRNA. The final total RNA concentration used was 2.5 ng/µL. The reaction was performed at 16 °C for 30 min, 42 °C for 30 min and 85 °C for 5 min. We used 1.33 µL of the obtained cDNAs in a subsequent quantitative qRT-PCR amplification using the TaqMan Universal PCR master mix (Applied Biosystems) and the associated specific probe provided in the TaqMan MicroRNA Assay Kit (Applied Biosystems). Specific Taqman probes were used for each gene as follows: microRNA-122-5p (miR-122-5p; hsa-mir-122-5p), 5′-UGGAGUGUGACAAUGGUGUUUG-3′; microRNA-130a-3p (miR-130a-3p; hsa-mir-130a-3p), 5′-CAGUGCAAUGUUAAAAGGGCAU-3′; microRNA-486-5p (miR-486-5p; hsa-mir-486-5p), 5′-UCCUGUACUGAGCUGCCCCGAG-3′ and microRNA-718 (miR-718; hsa-mir-718), 5′-CUUCCGCCCCGCCGGGCGUCG-3′. The results were normalized to the expression of the U6 small nuclear RNA (U6 snRNA), which was used as an endogenous control. Amplification was performed using the ABI Prism 7300 SDS Real-Time PCR system (Applied Biosystems, Madrid, Spain) at 95 °C for 10 min, followed by 40 cycles of 95 °C for 15 s and 60 °C for 1 min. The fold change in the miRNA level was calculated by the log 2 scale according to the equation 2−ΔΔCt, where ΔCt = Ct miRNA-Ct U6 and ΔΔCt = ΔCt treated samples − ΔCt untreated controls.

### Functional and pathway enrichment analysis of the miRNA target genes

The target genes of miR-122, miR-130a and miR-486 were predicted using DIANA-miRPath (v3; microrna.gr/tarbase) [21], mapped on the Kyoto Encyclopaedia of Genes and Genomes (KEGG) pathways based on an enrichment analysis, and prioritized for the intracellular signal transduction pathways that could be regulated by the three-miRNAs.

Because of the limited target genes identified for miR-718, for this miRNA, we combined the targets genes predicted by four databases, namely, the TargetScan (v7.0; targetscan.org) [22], miRTarBase (2016 version; mirtarbase.mbc.nctu.edu.tw) [23], DIANA-microT-CDS (v3; microrna.gr/microT-CDS) [21] and miRDB (2016 version; mirdb.org) [24] databases. In this case, the functional classification of the target genes was carried out with Gene Ontology (GO) and pathway analysis using DAVID (david.ncifcrf.gov) [25] and WebGestalt (webgestalt.org) [26], respectively.

Cytoscape version 3.0.1 [27] was used to visualize the predicted miRNA-mRNA interaction. In the network, the intracellular signal transduction pathways are represented by hexagons-shaped nodes, the miRNAs are represented by rectangle-shaped nodes and the target genes are represented by round-shaped nodes.

### Statistical analysis

The means of two groups were compared using Student’s t test. A Chi squared test or a Fisher’s exact test, when appropriate, was used to compare the frequencies of the categorical variables. The effects of the valve morphology and aortic dilation were assessed using analysis of variance (ANOVA). Tukey’s test was used for the pairwise comparisons. A Pearson’s correlation was used to identify linear relationships between the miRNA expression levels and the diameter of the ascending aorta. Backward linear regression models were used to identify the independent predictors of the expression of the circulating miRNAs. p values <0.05 were considered significant. The statistical analyses were performed using SPSS software version 21.0 (IBM, Chicago, IL, USA).

## Results

### Identification of the circulating miRNome profiles

**Fig. 1:**
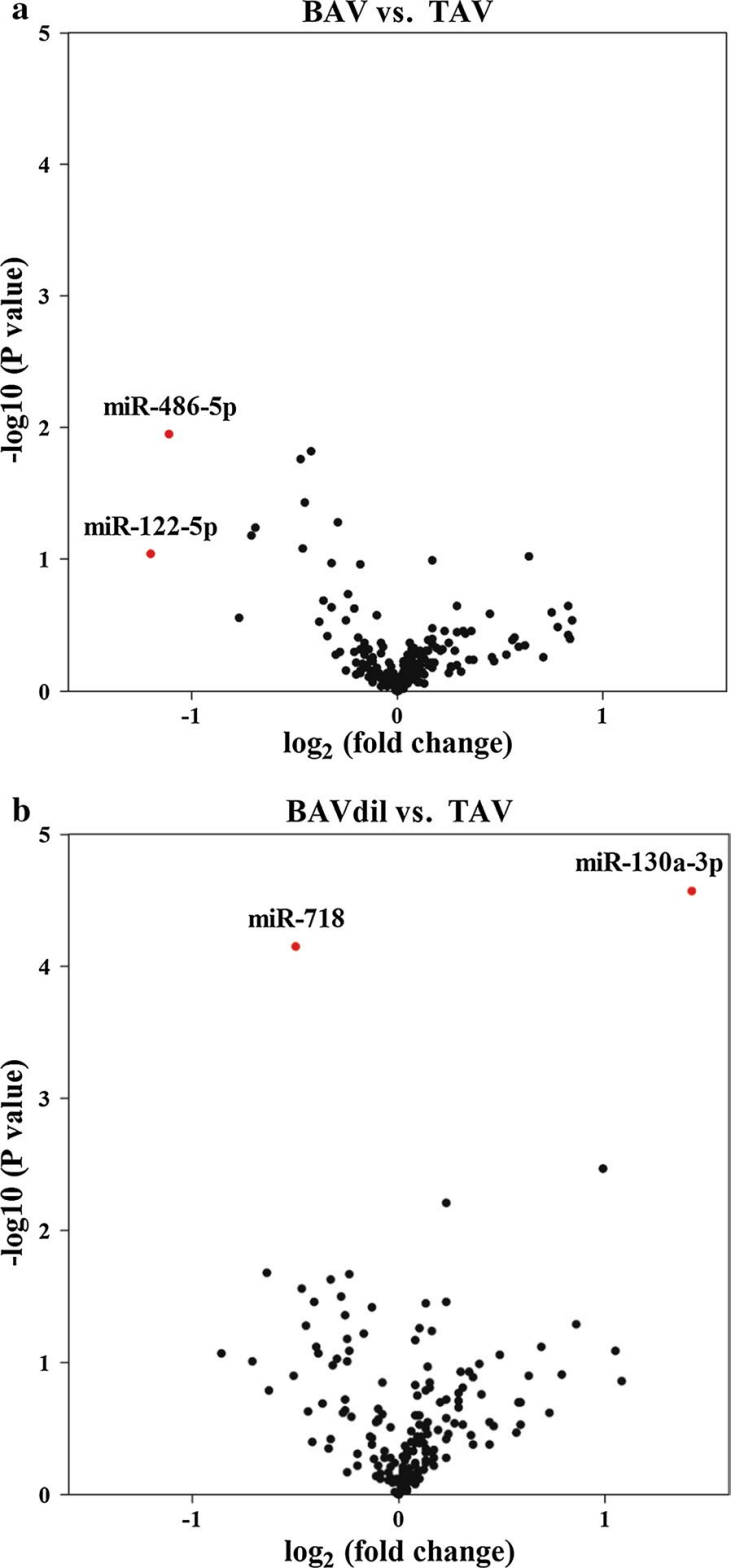
Volcano plot representing differences in the expression levels of the circulating miRNAs depending on **a** the bicuspid or tricuspid morphology of the aortic valve and **b** the morphology of the valve together with the dilation of the aorta. The log-fold change is plotted against the −log10 (p value)

Global miRNA expression profiling was performed on the plasma samples of the TAV controls and the BAV_non-dil_ and BAV_dil_ patients to identify the miRNA candidates, and the expression of these miRNAs was altered in BAV because of the valve morphology and/or because of the aortic dilation. The clinical characteristics of the patients involved in this stage are provided in Table 1. After processing the microarray data corresponding to the miRNA expression profiles, 260 miRNAs were identified as expressed in at least 10% of the samples. We performed a paired comparison between the groups to identify miRNA candidates for not only BAV but also aortic dilation. In this manner, we first analysed the effect of the valve morphology (comparing patients with TAV_non-dil_ and BAV_non-dil_) on the miRNome, and as shown in the volcano plot (Fig. 1a), the expression levels of miR-122 and miR-486 differed significantly from the remainder, indicating that the expression levels of these two miRNAs in plasma might be associated with the morphology of the aortic valve.

In a second analysis, we compared the two most divergent groups (TAV_non-dil_ and BAV_dil_) to integrate the complexity associated with the valve morphology and the dilation of the ascending aorta. This analysis indicated that the expression of two other miRNAs, miR-130a and miR-718 (Fig. 1b), was significantly different between the plasma of the TAV_non-dil_ and BAV_dil_ patients.

### RT-qPCR validation of the miRNA candidates in an independent cohort

The miRNA candidates selected using the microarray approach were validated by RT-qPCR in a new set (n = 43), which comprised TAV_non-dil_ healthy controls and BAV_non-dil_ and BAV_dil_ patients, and we also included a new group of patients with TAV and aortic dilation (TAV_dil_) to determine the specific pattern associated with the valve morphology or aortic dilation *per se*.

#### The expression levels of miR-122, miR-130a and miR-486 are associated with the morphology of the aortic valve

**Fig. 2:**
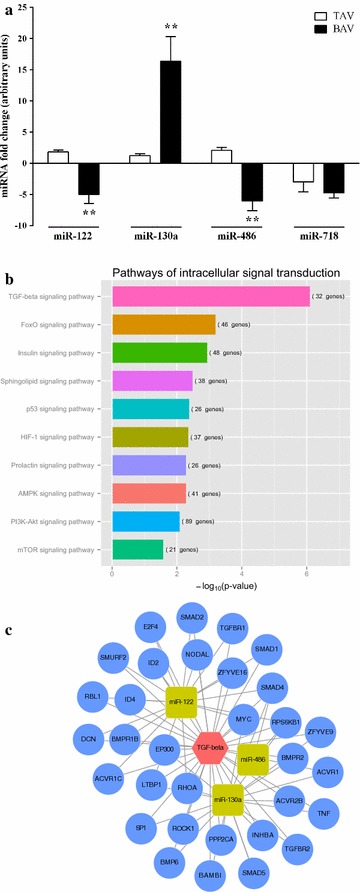
The expression levels of miR-122, miR-130a and miR-486 are influenced by the morphology of the aortic valve. **a** The validation of the miRNA candidates for aortic morphology was performed by RT-qPCR. *Two asterisks* Significant values (p < 0.001; unpaired t test). **b** The pathways of intracellular signal transduction were significantly enriched by the predicted target genes of miR-122, miR-130a and miR-486. **c** Interactive miRNA-gene network regarding the TGF-beta signalling pathway. *TAV* tricuspid aortic valve, *BAV* bicuspid aortic valve, *TGF* transforming growth factor

We compared the plasma expression of the miRNA candidates, miR-122, miR-130a, miR-486 and miR-718, which depended on the morphology of the valve and was independent of the presence or absence of dilation in the ascending aorta (Fig. 2a).

This validation study corroborated that the expression of three of the miRNA candidates, miR-122, miR-130a, and miR-486, differed significantly between the plasma of TAV individuals and that of BAV patients (BAV vs. TAV), and therefore, their expression levels in the circulation were influenced by the morphology of the valve. The bicuspid valve morphology was associated with decreased circulating levels of miR-122 and miR-486 instead of the increased levels reported for miR-130a.

We performed a functional categorization of the miRNA target genes to gain further insight into the biological role of miR-122, miR-130a and miR-486 in BAV (Fig. 2b). The targets of each of the selected miRNAs were predicted, mapped on KEGG pathways based on an enrichment analysis, and used to prioritize the intracellular signal transduction pathways that could be regulated by the three miRNAs. Using this approach, we identified the transforming growth factor-beta (TGF-β_1_) signalling pathway as the most significantly enriched pathway (p = 7.73 × 10^−7^), with a total of 32 genes targeted by the four selected miRNAs (Fig. 2c).

#### Effect of aortic dilation on the miR-130a and miR-718 expression levels in plasma

**Fig. 3:**
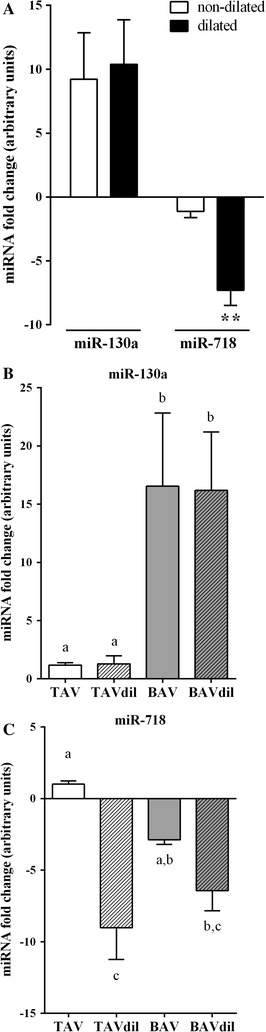
The expression of circulating miR-718 is affected by the dilation of the ascending aorta. **A** RT-qPCR validation of the effect of the ascending aortic dilation on the expression levels of miR-130a and miR-718 in the plasma. The specific effects of the valve morphology and aortic dilation were analysed for miR-130a (**B**) and miR-718 (**C**). *Two asterisks* Significant values (p < 0.001; unpaired t test). The *bars* with different letters are significantly different (one-way ANOVA, Tukey test, p < 0.05)

Next, we validated whether the modulation of the miRNA expression profile of miR-130a and miR-718 in the plasma was related to the dilation of the aorta. We first analysed the effect of the aortic dilation on miR-130a and miR-718, comparing the expression levels between the dilated and non-dilated patients independent of the aortic valve morphology (n = 43; Fig. 3A). The up-regulation of miR-130a expression in the plasma of the BAV patients was only influenced by the morphology of the aortic valve, and the dilation of the aorta had no modulatory effect. In contrast, the plasma expression of miR-718 was significantly affected by the dilation of the ascending aorta, and this dilation-promoted effect was masking the differential expression consequence of the valve morphology not observed in the comparisons used in the previous validation.

We explored these morphology- and aortic-dilation-mediated effects by comparing the expression of miR-130a (Fig. 3B) and miR-718 (Fig. 3C) between the four sub-groups depending on the valve morphology and the dilation of the ascending aorta (TAV_non-dil_, TAV_dil_, BAV_non-dil_ and BAV_dil_). This analysis corroborated that up-regulated miR-130a expression was associated only with the bicuspid morphology of the aortic valve, and no effect of the aortic dilation was observed. Furthermore, the down-regulated expression of miR-718 observed in BAV patients was significantly more prevalent in the presence of aortic dilation compared with the expression in the non-aortic dilated patients and was independent of the tricuspid or bicuspid aortic valve morphology.

### The expression of miR-718 in plasma is a predictor of aortic dilation

**Fig. 4:**
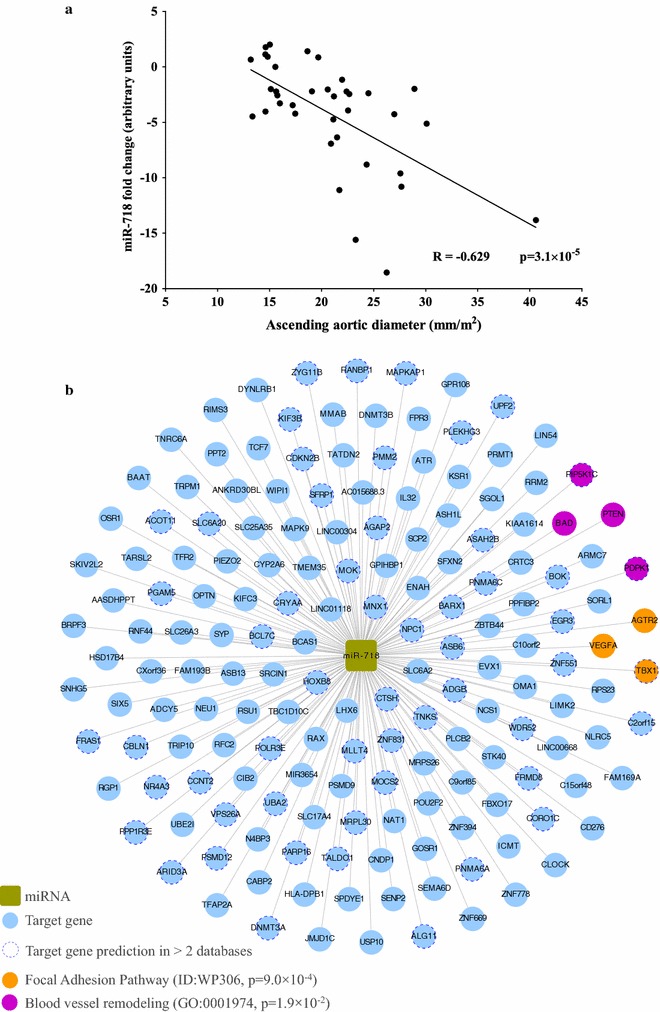
Functional implications of miR-718 expression in ascending aortic dilation. **a** The expression of circulating miR-718 is inversely correlated with the diameter of the ascending aorta. **b** miR-718 target gene network resulting from the functional enrichment analysis

The role of miR-718 as a biomarker of aortic dilation was supported by the inverse correlation between miR-718 plasma expression and the diameter of the ascending aorta (R = −0.629, p = 3.1 × 10^−5^; Fig. 4a) in all of the patients in the validation cohort; this correlation was independent of valve morphology (TAV and BAV) or the presence or absence of aortic dilation (dilated and non-dilated). Furthermore, using this validation cohort, we built a multiple logistic and linear models using the miR-718 expression profiles as the predictive variables and the dilation of the aorta (represented as a categorical variable or as the diameter of the aorta) as the outcome variable in addition to age and hypertension as possible confounding factors (Table 3). This analysis confirmed that the expression of miR-718 in the plasma is an independent predictor of the dilation of the aorta.

**Table 3:** Logistic and linear regression analyses of the miR-718 expression levels as a predictor of aortic dilation

	Aortic dilation		Indexed aortic diameter (×mm/m^2^)	
	β	p	β	p
Age (years)	0.317	0.118	0.234	0.177
Hypertension	0.050	0.801	0.301	0.078
miR-718 expression	−0.407	0.022*	−0.541	0.002*

The analysis was performed in the independent validation cohort, including TAV, TAVdil, BAV and BAVdil (n = 43)

* Significant values (p < 0.05)

To provide a better understanding of the possible functional relevance of miR-718 down-regulation, we characterized the putative biological functions of this miRNA based on the identification of the miR-718 target genes. The predicted miRNA-gene interaction network (Fig. 4b) was composed of miR-718 and the 167 target genes. We also performed a functional enrichment analysis to predict the potential biological function of the miR-718 target genes. DAVID was used to determine the GOs associated with biological processes that might be overrepresented within the miR-718 target genes. Four of the target genes were significantly enriched for the GO biological process “blood vessel remodelling” (p = 1.9 × 10^−2^). Using WebGestalt, we performed a functional analysis based on the pathways that might be associated with the miR-718 targets; 3 of the miR-718 target genes were significantly enriched for the “focal adhesion pathway” (p = 9.0 × 10^−4^).

## Discussion

This study found that the miRNA expression pattern in plasma is influenced by the morphology of the aortic valve and the dilation of the ascending aorta. Using comprehensive miRNA expression profiling and further RT-qPCR validation, we identified a circulating miRNA signature, including miR-122, miR-130a and miR-486, whose regulation is dependent on the tricuspid or bicuspid morphology of the aortic valve. Furthermore, the plasma expression of miR-718 may be a biomarker of aortic dilation, that is, an increased ascending aorta diameter is associated with the down-regulation of miR-718. We explored the biological implications of the differential expression of these miRNAs based on target gene prediction and the putative biological functions and processes regulated by the target genes. In this manner, the differential modulation of the bicuspid valve morphology-associated miRNAs might affect the TGF-β_1_ signalling pathway, and the dysregulation of miR-718 might be associated with the progression of aortic dilation by modulating the focal adhesion and blood vessel remodelling processes.

The molecular features and mechanisms that underlie BAV and the dilation of the aorta are poorly understood, and to our knowledge, the miRNA expression profile and putative gene regulation in BAV disease and aortic dilation identified in this study by screening in plasma of BAV patients has not been previously reported. Previous studies have analysed the expression of miRNAs in aortic tissue segments of patients with BAV and animal models. Nigam et al. [28] determined that miR-26a, miR-30b and miR-195 were decreased in the aortic valve leaflets of patients requiring aortic valve replacement due to aortic stenosis compared to those requiring replacement due to aortic insufficiency, and both pathologies are associated with BAV. Using porcine valvular interstitial cells and human valve leaflets, Yanagawa et al. [29] determined that the down-regulation of miR-141 in stenotic bicuspid leaflets regulates BMP-2-mediated valve calcification. Wu et al. [30] performed a paired comparison of the miRNA expression between severely dilated and normal-appearing, less-dilated aortic samples, associating the differential regulation of the miR-17 gene cluster with the predisposition to dilation through the dysregulation of the matrix metalloproteinases-tissue inhibitors of matrix metalloproteinases (TIMP-MMP) pathway. However, although the determination of the miRNA expression profile in tissue samples provides valuable information regarding the pathophysiological mechanisms underlying diseases, this expression signature often cannot be extrapolated to the bloodstream. Thus, unlike these tissue-specific miRNAs, the determination of a circulating miRNA expression profile allows for the integration of the complexity associated with multiple tissues, as is in the case of complex diseases, in addition to serving as biomarkers of these diseases.

In clinical practice, the diagnosis of BAV and ascending aorta dilation is currently performed exclusively based on imaging techniques (i.e., echocardiography, TAC, and magnetic resonance) and the subsequent interpretation by an expert. Moreover, both BAV and the dilation of the aorta are normally presented as asymptomatic clinical conditions, and the molecular causes underlying both of these pathologies are unknown. Therefore, determining plasma molecular biomarkers of BAV and the progression of aortic dilation would be extremely useful in expanding the basic knowledge of the aortic dysfunction, as well as for the identification of new therapeutic targets that might significantly improve the diagnosis, prognosis and treatment of BAV and aortic dilation. Moreover, the determination of biomarkers in the plasma has an added value because in the plasma, the miRNA levels are reproducible and consistent among individuals, and the miRNAs appear to be protected from endogenous ribonuclease-induced degradation [31]. In addition, in contrast to other tissues, the plasma is more easily accessible by non-invasive means, which might improve the feasibility of these molecules as useful biomarkers in routine clinical practice.

The bicuspid morphology of the aortic valve is associated with an abnormal blood flow and shear stress at the ascending aorta that leads to damage and apoptosis of endothelial cells at the cellular level, involving the activation of multiple biological processes, such as endothelial dysfunction, inflammation, oxidative stress, angiogenesis and extracellular matrix remodelling [32–34]. Considering this BAV-associated environment, we hypothesized that circulating miRNAs might be involved in the paracrine communication between cells and contribute to the regulation of disease progression, and thus, the circulating levels of miRNAs might reflect the hemodynamic alteration of the blood flow resulting from the bicuspid morphology of the aortic valve.

In addition to their role as biomarkers for various diseases, miRNAs can be considered potential dynamic master regulators of signalling pathways due to their capacity to specifically control the expression of key components of signal transduction at multiple levels. Therefore, based on a functional analysis, our data supported the crucial role of the dysregulation of genes involved in the TGF-β_1_ signalling pathway in BAV disease [35]. In addition, we propose that miR-122, miR-130a and miR-486 might serve as molecular effectors in the BAV associated dysregulation of TGF-β_1_ receptor. TGF-β_1_ is a crucial factor mediating tissue fibrosis and extracellular matrix (ECM) remodelling in ascending aorta. TGF-β_1_ is a pleiotropic cytokine secreted and stored in the ECM as a biologically inactive form that is complexed with latent TGF-β_1_ binding protein (LTBP-1) in the ECM. Activated TGF-β_1_ binds to a heterodimeric receptor complex comprising two subunits (TGFβR1 and TGFβR2) whose genes are targeted by miR-122 and miR-130a, respectively, activating Smad proteins, which mediates the intracellular signalling of TGF-β_1_ [36]. The phosphorylation of SMAD2 (the gene targeted by miR-122) and SMAD3 results in the formation of heterooligomeric complexes with SMAD4 (the gene targeted by miR-122, miR-130a and miR-486). TGF-β_1_ signalling has been reported to play divergent roles in aortic dilation depending on its location (i.e. abdominal or thoracic aortic aneurysms) or aortic valve morphology [37]. As limitation of this study, the role of these miRNAs in the regulation TGF-β1 signalling pathway was determined based on computational predictions and it was not experimentally confirmed.

We can speculate that the opposite regulation of mir-122 (decreased expression levels) and miR-130a (increased expression levels) that we observed could be associated with the imbalance of TGFβR1 and TGFβR2 subunits. This speculation is supported by previous findings where the postnatal disruption of the *Tgfbr2* gene expression was associated with decreased canonical TGF-β_1_ signalling and accelerated aneurysm growth in a murine model of Marfan syndrome [38]. Moreover, Forte et al. [39], reported that ascending aorta aneurysms in BAV patients was associated with an imbalance between the TGFβR1 and TGFβR2 subunits, although, in that case the authors associated this imbalance by an intrinsic defect of TGFβR1 expression. This imbalance between both subunits of the TGF-β_1_ receptor could be indicating a redirection towards the activation of the non-canonical TGF-β_1_ signalling [37], which is related to the induction of the ECM degradation and MMP activity, both biological processes associated with BAV and aortic dilation.

In addition to the biological and cardiology-relevant implication of each miRNA individually, miR-122 accounts for 70% of the total miRNAs in the adult liver [40] and is considered a central player in liver biology and in the regulation of cholesterol and fatty acid metabolism [41]. Interestingly, Beaumont et al. [42] demonstrated that the down-regulation of miR-122 was associated with the up-regulation of TGF-β_1_ expression, increasing the severity of myocardial fibrosis in patients with aortic stenosis, which is the most frequent valvular complication in BAV patients [43]. miR-130a has important roles in the cardiovascular system due to its capacity to promote the proliferation of endothelial cells and vascular smooth muscular cells. In fact, miR-130a has been described as a regulator of the angiogenic phenotype of vascular endothelial cells through its ability to modulate the expression of the homebox genes GAX and HOXA5 [44]. Furthermore, the expression of miR-130a in the aorta is correlated with vascular remodelling in spontaneously hypertensive rats (SHRs) [45]. Regarding miR-486, Holliday et al. [46] demonstrated that the expression of miR-486 in human aortic valvular endothelial cells was shear dependent, and Navon et al. [47] suggested that a reduction of miR-486 supports tissue remodelling characterized by increased proliferation. Therefore, our findings regarding the miRNA signature associated with the morphology of the aortic valve support the notion that miR-122, miR-130a and miR-486 play a key role in BAV disease and add new knowledge regarding the molecular effectors of the TGF-**β**_**1**_ pathway dysregulation associated with BAV.

We propose miR-718 as a new non-invasive biomarker of dilation of the ascending aorta. We demonstrated its potential as molecular feature associated with an aortic dilation in patients with a dilated ascending aorta that was independent of the tricuspid or bicuspid morphology of the aortic valve. We found only 4 references [48–51] in PubMed when searching in the literature for information on the biological function of miR-718; to date, no one has referred to the biological function of miR-718 expression in the cardiovascular field, including its function in relation to aortic valve morphology or the dilation of the aorta.

Using a bioinformatics approach, we predicted the putative target genes of miR-718 and the biological function and processes that it might regulate. The miR-718 target genes were significantly enriched for the “blood vessel remodelling” process and “focal adhesion pathway”. Ascending aorta dilation is defined as a degenerative vascular disorder due to the destructive remodelling of the aortic wall and the degradation of the ECM proteins, leading to the recruitment and infiltration of immune system cells mediated by the secretion of adhesion molecules. Interestingly, vascular endothelial growth factor A (VEGFA) is one of the miR-718 target genes that was significantly enriched for the “focal adhesion pathway”. VEGFA acts on endothelial cells by increasing vascular permeability and angiogenesis, inducing vasculogenesis and endothelial cell growth, promoting cell migration, and inhibiting apoptosis [52]. These biological implications of the miR-718 target genes might explain the relation between miR-718 and ascending aorta dilation.

In BAV, as in other disease-associated complications, the emergence of the ascending aorta dilation cannot be considered as a discrete alteration in time; instead, it is a continuous process associated with the pathophysiological implications of BAV, and such a complex process involves multiple cell signalling pathways that might be integrated to reflect the complexity of the disease. In this manner, our results are consistent with previous findings that postulated the crosstalk between TGF-β_1_ and VEGF together with the Notch signalling pathways, to act coordinately in space and time in the regulation of vascular morphogenesis [53]. With this consideration, our circulating miRNA signature, including miR-122, miR-130a, miR-486 and miR-718 expression, integrates the complexity associated with not only the bicuspid morphology of the aortic valve but also the progressive dilation of the ascending aorta. Furthermore, whether these miRNAs are directly involved in the aortic wall pathogenesis of BAV disease or if they are a consequence of the increased aortic shear stress generated by the anomalous aortic flow caused by BAV remains unknown.

## Conclusions

We proposed miR-122, miR-130a, miR-486 and miR-718 as new molecular features associated with BAV and aortic dilation by the regulation of TGF-β_1_ and vascular remodelling mediated by the VEGF signalling pathway.

## Abbreviations

TAV: tricuspid aortic valve
TAVnondil: tricuspid aortic valve with non-dilated aorta
BAV: bicuspid aortic valve
BAVnondil: bicuspid aortic valve with non-dilated aorta
miRNAs: microRNAs
LV: left ventricular
WSS: wall systolic stress
MMP: matrix metalloproteinases
TIMP: inhibitors of matrix metalloproteinases
ECM: extracellular matrix
TGF-β_1_: transforming growth factor-beta
VEGFA: vascular endothelial growth factor A
